# Association of alpha A-crystallin polymorphisms with susceptibility to nuclear age-related cataract in a Han Chinese population

**DOI:** 10.1186/s12886-017-0529-9

**Published:** 2017-07-29

**Authors:** Zhennan Zhao, Qi Fan, Peng Zhou, HongFei Ye, Lei Cai, Yi Lu

**Affiliations:** 1 Department of Ophthalmology, Eye and ENT Hospital of Fudan University, 83 Fenyang Road, Shanghai, 200031 People’s Republic of China; 2 Department of Ophthalmology, Parkway Health Hong Qiao Medical Center, Shanghai, 200336 People’s Republic of China

**Keywords:** Alpha A-crystallin, Age-related cataract, 5′ untranslated region, Single nucleotide polymorphisms, Transcriptional activity

## Abstract

**Background:**

Alpha A-crystallin (CRYAA) is considered critical for the maintenance of lens transparency and is related to the pathogenesis of age-related cataracts (ARCs), especially the nuclear subtype. As the 5′ untranslated region (5′ UTR) modulates gene expression, the purpose of current study was to investigate whether single nucleotide polymorphisms (SNPs) in the 5′ UTR of *CRYAA* were associated with susceptibility to ARC in a Han Chinese population and to clarify the mechanism of this association.

**Methods:**

SNPs in the 5′ UTR (−1 to −1000) of *CRYAA* were identified in 243 nuclear ARC patients and 263 controls using polymerase chain reaction and DNA sequencing. Allele and genotype frequencies were calculated and compared between two groups. Haploview 4.2 was used to calculate the linkage disequilibrium index, and the SHEsis analysis platform was used to infer haplotype construction. A dual-luciferase reporter gene assay was used for transcription of *CRYAA* in the presence of a protective haplotype with individual SNP alteration, Chromatin immunoprecipitation (ChIP) was employed to determine whether SNPs regulated *CRYAA* expression by altering the binding affinity of transcription factors.

**Results:**

Three polymorphisms were identified in the 5′ UTR of CRYAA: rs3761381 (*P* = 0.000357, odds ratio [OR] = 1.837), rs13053109 (*P* = 0.788, OR = 1.086), and rs7278468 (*P* = 0.00136, OR = 0.652). The haplotype C-G-T (*P* = 0.0014, OR = 1.536) increased the risk of nuclear ARC, whereas the haplotype T-G-G (*P* = 0.00029, OR = 0.535) decreased the risk. The haplotype C-G-T decreased CRYAA transcription through rs7278468, which is located in the binding site of specificity protein 1 (Sp1). Furthermore, the G allele of rs7278468 increased CRYAA transcription by enhancing the binding affinity of Sp1.

**Conclusions:**

These data indicate that the CRYAA polymorphism is a genetic marker of inter-individual differences in the risk of nuclear ARC.

**Electronic supplementary material:**

The online version of this article (doi:10.1186/s12886-017-0529-9) contains supplementary material, which is available to authorized users.

## Background

Age-related cataracts (ARCs), which are a major cause of blindness worldwide, are characterized by lens opacities and visual impairment due to degenerative changes in the lens in the elderly [1]. The causes of ARCs are multifactorial, with both environmental and genetic variations implicated in the disease [2]. A study of twins strongly implicated genetic factors in the pathogenesis of ARCs, demonstrating heritability of 48% for the nuclear subtype [2]. Recent studies also indicated that several genes, such as galactokinase and eph-receptor tyrosine kinase-type A2 [3, 4], were genetic risk factors for cataracts. Although the importance of genetic risk factors for ARC has been highlighted, the pathophysiology is far from clearly understood.

As a major structural protein component expressed in the lens, alpha A-crystallin (CRYAA) is considered critical for the maintenance of lens transparency [5]. Many studies showed that CRYAA was related to the pathogenesis of ARC, including a study conducted by our research group [6–8]. The chaperone-like activity of CRYAA enables it to protect other crystallins against thermally induced inactivation or aggregation [9]. In addition, CRYAA can trap aggregation-prone denatured proteins, an action that is thought to delay the development of ARC [10]. Although previous research demonstrated that the levels of CRYAA decreased in the nuclear capsule of ARC patients compared to those of controls [8], the mechanism underlying the downregulation of CRYAA in the lens was unclear.

Previous research demonstrated that the 5′ untranslated region (5′ UTR) acted as a regulatory element in genes and that it was associated with modulating the expression of gene-coding regions [11]. Single nucleotide polymorphisms (SNPs) in 5′ UTR sequences were shown to play a critical role in regulating gene expression [12]. In addition, previous studies showed that SNPs in the 5′ UTR of the *SLC16A12* gene were involved in the pathogenic consequences of ARC [13]. Recently, SNPs that were identified in the CRYAA gene promoter region that were associated with cortical ARC in a Northern Italian population [14]. However, it is unknown whether SNPs in the 5′ UTR of *CRYAA* contribute to ARC susceptibility in a Han Chinese population, especially the nuclear subtype (the most frequent form).

The present study focuses on polymorphisms within the 5′ UTR of CRYAA (−1 to −1000) and attempts to shed light on the development of nuclear ARC in a Han Chinese population.

## Methods

### Subjects

Subjects were recruited from the Eye and ENT Hospital of Fudan University. All the subjects underwent a full ophthalmic examination, including visual acuity, slit-lamp microscopic examinations, and ophthalmoscopic examinations. There was no consanguinity between the subjects (at least not among all four grandparents). All the enrolled subjects self-identified as Han Chinese (all four grandparents were ethnic Han Chinese). This research was approved by the Institutional Review Board and followed the tenets of the Declaration of Helsinki. All the subjects signed informed consent forms.

### Lens opacity grading

After pupil dilation with 1% tropicamide, a trained ophthalmologist graded the lens opacity of each right eye according to the Lens Opacity Classification System (LOCS) III. Opacity was classified as nuclear opalescence (NO), nuclear color (NC), cortical (C), and posterior subcapsular (P). The grading consisted of six standards for NO and NC (standards 1 to 6) and five standards for C and P (standards 1 to 5). Each standard was assigned using decimals to interpolate between the reference standards, with the assigned scores ranging from 0.1 to 6.9 for NO and NC and 0.1 to 5.9 for C and P. The subtype of the cataract was then classified as nuclear (NO or NC ≥3), cortical (C ≥ 2), posterior subcapsular cataract (PSC) (*P* ≥ 2), or mixed type (i.e., the presence of more than one type in one eye).

### ARC group and control group

All subjects with nuclear cataract were enrolled. Subjects with cortical and posterior subcapsular cataracts were excluded from this study. For the mixed type, only subjects with NO or NC scores higher than C and P scores were enrolled. Cases and controls were then recruited based on their NO and NC grading scores. The cases included subjects with NO and NC grading scores ≥3.0, and the controls included those with NO and NC grading scores <2.0.

The exclusion criteria for the cases and controls included: (1) subjects younger than 45 years; (2) pseudophakia or aphakia in either eye; (3) the presence of other eye diseases, such as dislocated lens, trauma, uveitis, high myopia, glaucoma, macular diseases, and retinal detachment; (4) previous ocular surgery in either eye; and (5) a history of diabetes, kidney disease, respiratory disease, cancer, or tumors.

### Blood sample collection and DNA isolation

Five milliliters of peripheral blood samples were collected in EDTA tubes from all the subjects was and stored at −80 °C until use. DNA was isolated from whole blood cells using a Mammal Blood Genomic DNA Extraction Kit (LifeFeng Biotech Co., Shanghai, China), following the manufacturer’s protocol, and was stored at −20 °C until used for genotyping.

### Identification and genotyping of SNPs

For analyzing the polymorphism in the 5′ UTR (−1 to −1000) of *CRYAA*, the polymerase chain reaction (PCR) and DNA direct sequencing were performed. A set of primers (forward: GGTGACACAGCAAGACTCCA and reverse: CACGTCCATGTTCAGCTTTG) from Generay Biotechnology Co., Ltd. (Shanghai, China) was used to amplify the target fragment. The PCR reactions were performed in 50 μl reaction mixtures, consisting of 25 μl of PrimeSTAR Max Premix (Takara), 20 μl of RNase-free water, 1 μl of the forward primer, 1 μl of the reverse primer, and 1 μl of extracted genomic DNA. The PCR program included a 94 °C activation step for 3 min, followed by 30 cycles of 94 °C for 20 s, 60 °C for 20 s, 72 °C for 40 s, and 72 °C for 65 s. The PCR products were sequenced using ABI 3730xl (Generay Biotechnology Co., Ltd).

### Cell culture and transfection

The human lens epithelium (HLE) B3 cell line obtained from American Type Culture Collection (ATCC; Rockville, MD, USA) was cultured in Dulbecco’s Modified Eagle Medium (DMEM) containing 20% fetal bovine serum (FBS). Before transfection, the cells were seeded in six-well plates and grown overnight, so that the cell density reached approximately 70%. A mixture of 2 μg of plasmid DNA and 4 μl of Lipofectamine™ 2000 (Invitrogen) reagent in 2 ml of serum-free medium was then added in a six-well plate. The cells were collected for luciferase activity testing and chromatin immunoprecipitation (ChIP) analysis 72 h and 48 h later, respectively.

### Plasmid construction

The human *CRYAA* 5′ UTR (−1 to −1000) was amplified using primers, as described above and then inserted into the PGL3-Basic vector (Invitrogen) using the restriction enzymes Hind III and KpnI (Takara). The *CRYAA* 5′ UTR containing each SNP mutation was constructed using site–directed mutagenesis, with the following primers: rs3761381-mut forward: ATCATGTGGGTGGTGGGTCT, reverse: ATGCCTTGATGTAACATCCCC; rs13053109-mut forward: GGTGAGACTCTGAGGACGATGTGT, reverse: ACACATCGTCCTCAGAGTCTCACC; rs7278468-mut forward: GGGTGTGTGCTCTCCCTCCTCT, reverse: AGAGGAGGGAGAGCACACACCC.

### Luciferase assay

The HLE cells were cultured in six-well plates with 2 μg of either PGL3-Basic-CRYAA 5′ UTR or PGL3-Basic plasmid and 20 ng of pRL-TK and then transfected into cells, as described above. Then, 72 h after transfection, luciferase activities were checked by a Dual-luciferase Reporter (DLR™) Assay System (Promega), according to the manufacture’s protocol.

### In silico analysis

The transcription factor binding sites of the identified SNPs of CRYAA 5’UTR were predicted by MEME SUITE as described previously [15].

### Chromatin immunoprecipitation (ChIP) analysis

ChIP analysis of HLE cells transfected with a plasmid containing the *CRYAA* 5′ UTR was conducted 48 h after transfection using a Pierce Agarose ChIP Kit (Thermo Scientific), according to the manufacture’s protocol. The antibodies used for ChIP included anti-human IgG (Abcam) and anti-specificity protein 1 (Sp1) (Abcam). The captured genomic DNA fragments were then purified with Premix Taq™ (Takara). The primers used for the ChIP PCR were as follows: CRYAA ChIP forward: CTGAGGACGATGTGTCTAACCTC, reverse: AGGCCTGGACTCAGCTGA. CRYAA ChIP NC: forward: ACCCTGACAGGAGCAGCCCA, reverse: TCCTCCAGGGGTCACATGC. The ChIP-quantitative PCR (qPCR) data relative to Sp1 were calculated using the 2^-ΔΔCt^ methods and presented as % input.

### Statistical analysis

Differences between the values were evaluated using a two-tailed Student’s *t*-test or Fisher’s chi-squared test, depending on the variables types. SPSS for Windows, version 17.0 (SPSS, IBM Inc., Chicago, IL, USA) was used in the statistical analysis of the above data and for the calculation of odds ratios (ORs). The Hardy–Weinberg equilibrium (HWE), linkage disequilibrium (LD) and the haplotype analysis was conducted using the SHEsis software platform [16]. The experiments were all repeated at least three times. A value of *P* < 0.05 was considered statistically significant.

## Results

### Participant characteristics

In total, 243 unrelated ARC patients and 263 control subjects were included in this study. The mean ages of the ARC patients and controls were 70.06 ± 6.22 years and 69.01 ± 4.64 years, respectively (*p* = 0.667).

### The SNPs identified in the CRYAA 5′ UTR

**Fig. 1:**
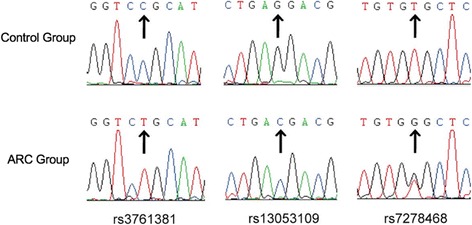
Sequence analysis of the 5’UTR region of *CRYAA* in the ARC group and the control group. The arrows point to the SNP sites

Three polymorphisms were identified in the 5′ UTR of CRYAA: rs3761381 (C > T), rs13053109 (G > C), and rs7278468 (T > G) (Fig. 1). The allele and genotype distributions of each identified SNP are shown in Table 1 and Table 2, respectively. All three SNPs were in Hardy Weinberg equilibrium (*P* > 0.05). The frequencies of the rs3761381 T allele (*P* = 0.000357, OR = 1.837,95% confidence interval [95% CI] = 1.312–2.572) and rs7278468 G allele (*P* = 0.00136, OR = 0.652,95% CI = 0.501–0.847) were significantly higher in the control group compared to the ARC group. With regard to rs13053109, there were no significant differences in the genotype or allele frequency between the ARC patients and the controls.

**Table 1:** Allele frequencies of SNPs in the CRYAA gene among the ARC group and the control group

SNPs	Allele	ARC group n (%)	Control group n (%)	*P* value	OR for minor allele (95% CI)
rs3761381				0.000357	1.837 (1.312–2.572)
	C	423 (87.0%)	413 (78.5%)		
	T	63 (13.0%)	113 (21.5%)		
rs13053109				0.788	1.086 (0.594–1.988)
	G	464 (95.5%)	504 (95.8%)		
	C	22 (4.5%)	22 (4.2%)		
rs7278468				0.00136	0.652 (0.501–0.847)
	T	344 (70.8%)	322 (61.2%)		
	G	142 (29.2%)	204 (38.8%)		

*SNPs* Single Nucleotide Polymorphisms, *ARC* Age-Related Cataract, *OR* Odds Ratio, *CI* Confidence Interval

**Table 2:** Genotype frequencies of SNPs in the CRYAA gene among the ARC group and the control group

SNPs	Genotype	ARC group n (%)	Control group n (%)	*P* value
rs3761381				0.0011
	C C	182 (74.9%)	161 (61.2%)	
	C T	59 (24.3%)	91 (34.6%)	
	T T	2 (0.8%)	11 (4.2%)	
rs13053109				0.965
	G G	222 (91.4%)	242 (92.0%)	
	G C	20 (8.2%)	20 (7.6%)	
	C C	1 (0.4%)	1 (0.4%)	
rs7278468				0.0054
	T T	120 (49.4%)	99 (37.6%)	
	T G	104 (42.8%)	124 (47.1%)	
	G G	19 (7.8%)	40 (15.2%)	

*SNPs* Single Nucleotide Polymorphisms, *ARC* Age-Related Cataract

### LD analysis and haplotype construction

**Fig. 2:**
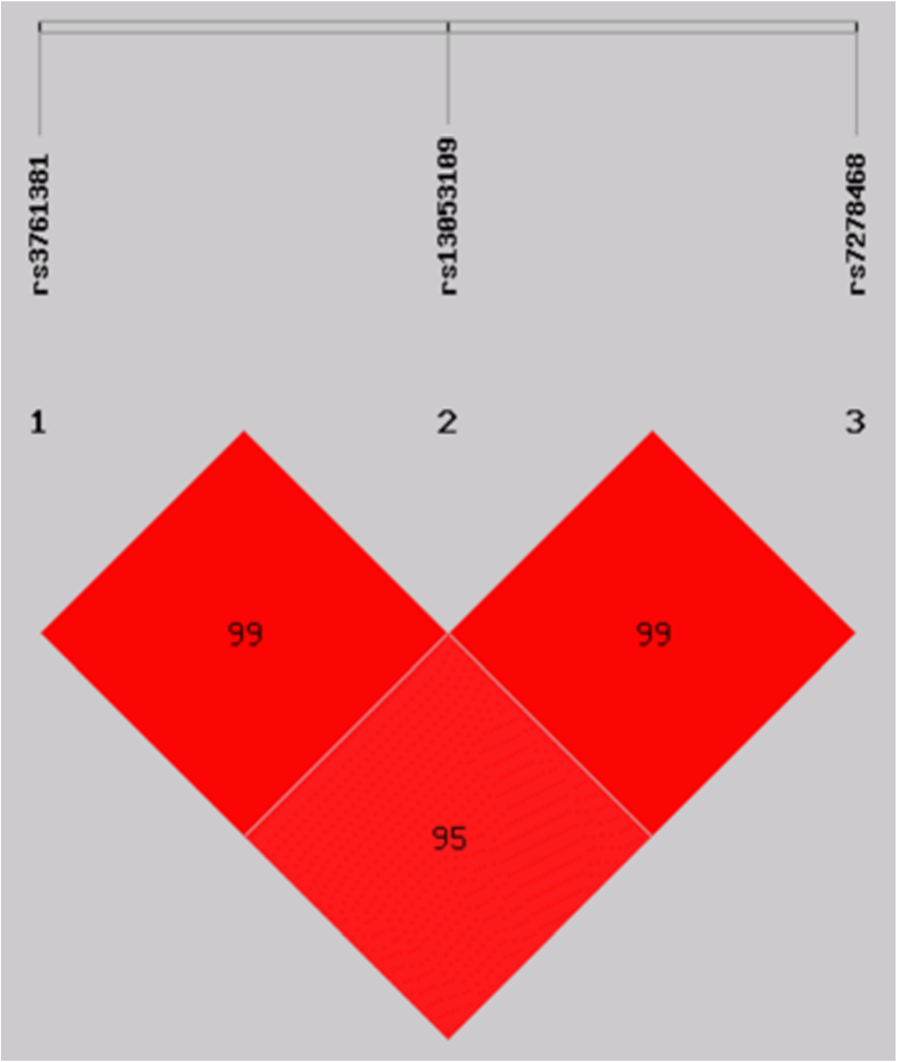
LD status of the three SNPs in *CRYAA 5’UTR*. Each square represents a pair-wise comparison between the 2 SNPs, and the respective D’ is given within each square. The darker colored squares indicate higher values of D’

The D’ values for the LD of the three SNPs are shown in Fig. 2 (D’ values: all >0.9). As seen, there were very strong levels of LD in all three SNPs. Five possible haplotypes were constructed (Table 3). The haplotype C-G-T appeared to confer a high risk of ARC (*P* = 0.0014, OR = 1.536, 95% CI = 1.180–1.997), whereas the haplotype T-G-G (*P* = 0.00029, OR = 0.535, 95% CI = 0.381–0.753) seemed to confer protection against ARCs.

**Table 3:** Haplotype analysis of the ARC group and the control group

Haplotypes	ARC group (freq)	Control group (freq)	*P* value	OR (95% CI)
C-C-G	22.00 (0.045)	22.00 (0.042)	0.788	1.087 (0.594–1.989)
C-G-G	59.31 (0.122)	71.32 (0.6136)	0.522	0.886 (0.613–1.282)
C-G-T	341.69 (0.703)	319.68 (0.608)	0.0014	1.536 (1.180–1.997)
T-G-G	60.69 (0.125)	110.68 (0.210)	0.00029	0.535 (0.381–0.753)
T-G-T	2.31 (0.005)	2.32 (0.004)	0.695	1.247 (0.816–1.457)

*ARC* Age-Related Cataract, *OR* Odds Ratio *CI* Confidence Interval

### The transcriptional activity of CRYAA in the presence of the haplotypes

**Fig. 3:**
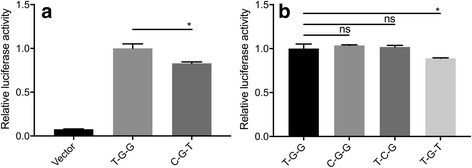
Allelic variation of the rs7278468 alters the transcription of CRYAA. **a** The luciferase activity of CRYAA 5’UTR within either the T-C-G or C-G-T haplotype after being transfected into HLE cells. **b** Site–directed mutagenesis was applied to discern the impact of rs3761381, rs13053109 and rs7278468. The allele rs7278468 is mainly accounted for the transcriptional activity alteration of the CRYAA 5’UTR. The CRYAA 5’UTR with T-C-G were set to 1, to which other values were normalized. The results are shown as the mean ± SD. (*p < 0.05)

Based on the position of these three SNPs, we constructed luciferase reporter vectors containing the CRYAA 5′ UTR and either T-G-G (protective haplotype) or C-G-T (risk haplotype) and evaluated whether they could influence the transcriptional activity of the gene. Seventy-two hours after transfection of the HLE cells, a dual-luciferase reporter assay showed that the luciferase activity of the cells transfected with the vector containing the CRYAA 5′ UTR (Fig. 3) was more than 11 times higher than that of the cells transfected with the vector alone, demonstrating that the CRYAA 5′ UTR exhibited positive transcriptional activity in the HLE cells. The transcriptional activity of the CRYAA 5′ UTR with the risk-associated haplotype C-G-T was approximately 17% lower than that of the protective T-G-G haplotype (Fig. 3a). This finding indicated that the C-G-T haplotype reduced CRYAA transcriptional activity in HLE cells and that this haplotype might cause nuclear ARC by decreasing the transcriptional efficiency of CRYAA.

### The transcriptional activity of *CRYAA* in the presence of the protective haplotype with individual SNP alteration

To distinguish which individual SNPs of the haplotypes in the CRYAA 5’ UTR were predominantly responsible for the regulation of transcriptional activity, site-directed mutagenesis was employed to alter the single base of each individual SNP present in the protective T-G-G haplotype, one by one. Subsequent transcriptional activity was then analyzed. As shown by a dual-luciferase reporter assay, 72 h after transfection, the transcriptional activities of the 5’ UTR containing the individual alleles of rs3761381 and rs13053109 occurring as a single base variation were not significantly different from those of the protective T-G-G haplotype (Fig. 3b). However, when the G allele of rs7278468 replaced with the T allele, the consequent transcriptional activity of the *CRYAA* 5′ UTR with the T-C-T haplotype decreased approximately 11% (*P* < 0.05) compared with that of the protective T-G-G haplotype. These data indicated that the allelic change in rs7278468 affected CRYAA expression.

### The rs7278468 G allele increased *CRYAA* transcription by enhancing the binding affinity of Sp1

**Fig. 4:**
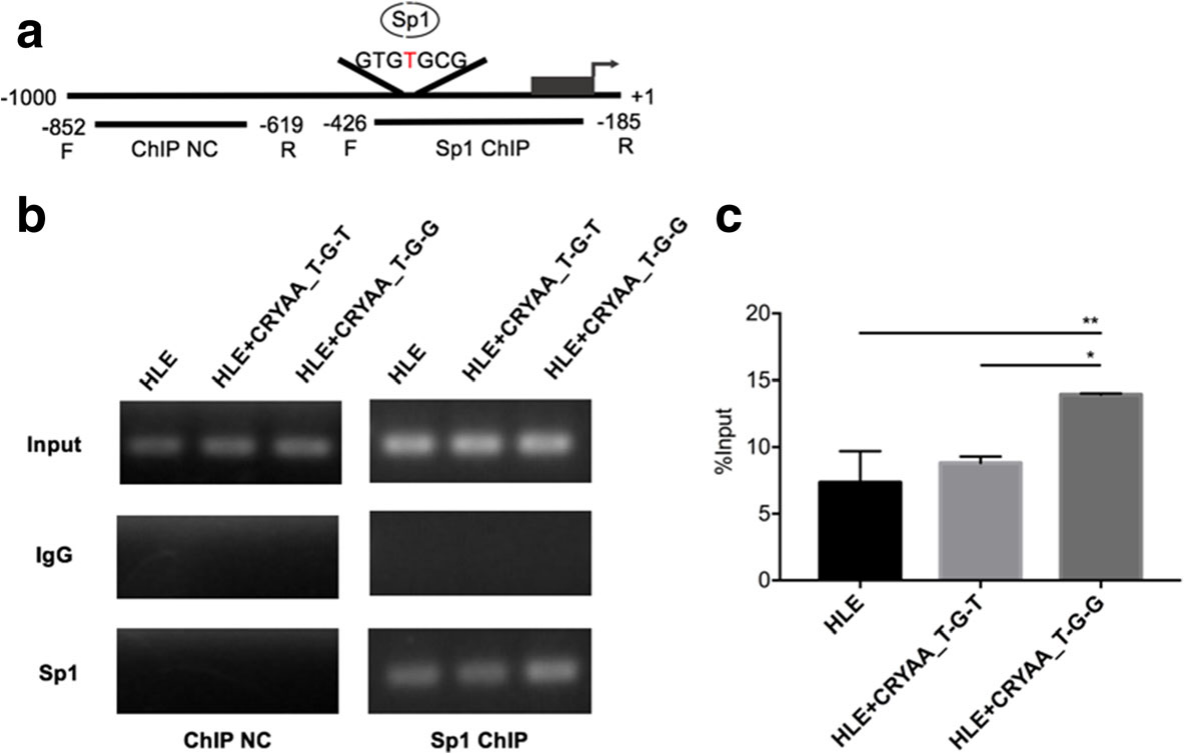
The rs7278468 G allele increases *CRYAA* transcription through enhancing Sp1 binding capacity. **a** Diagram of the CRYAA 5’UTR shows the Sp1 binding site within rs7278468. **b** ChIP analyses showing the rs7278468 G allele enhanced Sp1 binding. Right panel shows Sp1 binding to its responsive elements; left panel represents the control, which shows the DNA beyond Sp1 binding sites. Additionally, input is the positive control for the total genomic DNA extract before precipitation, and IgG is the negative control for nonspecific binding. A stronger PCR band was detected in the *CRYAA*_T-G-G transfected cells. **c** Levels of HLE cells alone and CRYAA_C-G-T and CRYAA_T-G-G transfected cells immunoprecipitated using Sp1 antibodies were measured following q-PCR. Signals from IgG controls used for each ChIP were subtracted from each of the Sp1 immunoprecipitation signals and the fold enrichment ratios of ChIP enriched vs total input were represented. The results are shown as the mean ± SD. (*p < 0.05). Gel photographs presented here are cropped and the originals are available in the Additional file 2: Figures S1 and S2)

Previous data showed that the rs7278468 alleles affected *CRYAA* transcriptional activity, as it lies upstream of the transcription start site. It is feasible that it might influence the binding affinity of one or more transcription factors and affect the transcriptional activity of *CRYAA*. Thus, using the MEME SUITE, transcription factor binding prediction of the sequence around rs7278468 was performed. The rs7278468 T allele is located in the binding motifs of several transcription factors, including specificity protein 1 (Sp1) (Additional file 1: Table S1). Sp1 is a transcriptional factor that applies its activity via binding to a GC-rich element in the promoter region of target genes. The methylation within the CpG sites of the CRYAA promotor might decrease the DNA-binding capacity of Sp1, leading to epigenetic repression in nuclear ARC lenses. The aforementioned factors make Sp1 a good candidate for further studies. To determine whether rs7278468 regulated *CRYAA* by altering the binding affinity of Sp1, ChIP-PCR was used to analyze the binding affinity of Sp1 in HLE cells (Fig. 4). With the ChIP-NC primers, bands were detected only in the input lanes and not in any of the immunoprecipitated samples. As shown by the ChIP-PCR, bands were detected in both the HLE cells alone and in the HLE cells transfected with the CRYAA_T-G-T or *CRYAA*_T-G-G plasmid. However, a higher strength ChIP-PCR band was detected in the *CRYAA*_T-G-G transfected cells (Fig. 4b), and the ChIP-qPCR confirmed this tendency (Fig. 4c). These data showed that Sp1 binds directly to the CRYAA 5′ UTR and that the rs7278468 G allele increased this binding. These findings implied that Sp1 might regulate the transcription of *CRYAA* by direct interaction with the 5′ UTR and that the rs7278468 G allele increased this interaction and enhanced transcriptional activity.

## Discussion

This study demonstrated evidence of the involvement of alleles, genotypes, and haplotypes of SNPs in the 5′ UTR of *CRYAA* in the susceptibility to nuclear ARC. Similar results were reported previously in the case of all types of ARC (especially the cortical subtype) in a northern Italian population [14]. However, in the Han Chinese population with nuclear ARC in the current study, the sites of the SNP (rs3761381) and allelic frequency (rs7278468) were significantly different than those reported. In the present study, the frequency of the T allele of rs7278468 was higher (70.8%) than that (49.5%) reported in a previous study of patients with ARC. Numerous studies have demonstrated a population-specific and subtype-specific effect of SNPs, and ARC is no exception [17–19]. The increased frequency of the T allele of rs7278468 observed in the present study might indicate an increased risk of the progression of ARC in the Han Chinese population, which was similar to epidemiological findings that the incidence rate of nuclear cataract in the Chinese population is higher than that in the Caucasian population [20, 21]. Furthermore, as rs7278468 has been found to be associated with cataracts in different populations, which points to the importance of the rs7278468 locus in the pathogenesis of cataracts, providing a potential target for future gene therapy. Additionally, the difference of allelic frequency between ours and Ma’s study might be caused by selection bias. They included all types of ARC, but our study included only nuclear ARC. Therefore, further studies with all types of ARC should be considered.

As a molecular chaperone, CRYAA protects other crystallins from aggregation or inactivation and to traps aggregation-prone denatured proteins, which are suggested to delay the progression of ARC [10]. Studies with CRYAA-knockout animals and our previous reports demonstrated the importance of CRYAA in lens clarity [7, 22]. In a previous study, we showed that CRYAA expression decreased in the lens capsules of individuals with age-related nuclear cataracts compared to age-matched controls and confirmed lower CRYAA levels in samples with greater lens opacity severity [8]. The decrease in CRYAA expression may be one cause of lens opacity and also contributes to cataract pathology.

SNPs in the 5′ UTR play a critical role in gene expression and are involved in the pathophysiology of disease. Seshadri identified a SNP, rs366316, in the 5′ UTR of CD1a that was strongly associated with CD1a expression [23]. SNPs identified in the 5′ UTR of the *SLC16A12* gene affected the pathogenic consequences of ARC [13]. As shown in the current study, Sp1 appeared to regulate *CRYAA* via binding to the binding motif in the 5′ UTR of CRYAA, with an increase in the binding strength of the Sp1 binding motif in the rs7278468 G allele increasing CRYAA transcription. The resulting increased in CRYAA transcription seemed to enhance the expression of the alpha A-crystallin protein in the lens, making individuals with the rs7278468 G allele invulnerable to the development of nuclear ARC.

Sp1 is a ubiquitously expressed transcription factor, which plays a critical role in regulating plenty of genes required for normal cell function [24, 25]. In human cells, SP1 acts as gene activator [25]. The DNA-binding affinity of Sp1 can be regulated both by altering protein interactions and by post-translational modification [26]. Recently, Liu reported that the methylation of CpG sites of the CRYAA promotor directly decreased the DNA-binding capacity of Sp1, leading to a reduction in the expression of CRYAA in HLE cells [27]. In the current study, Sp1 bound directly to the CRYAA 5′ UTR, and the rs7278468 G allele increased the binding affinity of Sp1, suggesting a mechanism for Sp1 regulation of CRYAA transcription.

To address the limitation of the sample size, larger sample studies are required to validate the association we have observed. Further studies could also be focused on the association of SNPs with cortical and PSC cataracts.

## Conclusions

This study identified three SNPs and their haplotypes in the 5′ UTR of CRYAA that appeared to be associated with nuclear ARC. As shown by the analysis of the effects of the individual SNP alleles on *CRYAA* transcription, rs7278468 appeared to be mainly responsible for the alteration in the transcriptional activity of the 5′ UTR of CRYAA. It exerted this effect through its G allele, which strengthened the binding affinity of Sp1. Manipulating these polymorphisms may provide a strategy to prevent or slow the progression of ARC.

## Additional files

Additional file 1: The transcription factor binding sites overlapping rs7278468. This is the raw data of the in-silico analysis. (DOCX 52 kb)

Additional file 2: Figures S1 and S2. The whole gels of the NC and Sp1 ChIPs respectively. This is the raw data of the chromatin immunoprecipitation (ChIP) analysis described above. (DOCX 645 kb)

## Abbreviations

5’UTR: 5′ untranslated region
ARC: Age-related cataract
C: Cortical
ChIP: Chromatin immunoprecipitation
CI: Confidence interval
CRYAA: Alpha A-crystallin
DMEM: Dulbecco’s Modified Eagle Medium
FBS: Fetal bovine serum
HLE: Human lens epithelium
HWE: Hardy–Weinberg equilibrium
LD: Linkage disequilibrium
LOCS: Lens Opacity Classification System
NC: Nuclear color
NO: Nuclear opalescence
OR: Odds ratio
PCR: Polymerase chain reaction
PSC: Posterior subcapsular cataract
SNPs: Single nucleotide polymorphisms
Sp1: Specificity protein 1

## References

[1] Congdon NG, Friedman DS, Lietman T (2003). Important causes of visual impairment in the world today. JAMA.

[2] Hammond CJ, Snieder H, Spector TD, Gilbert CE (2000). Genetic and environmental factors in age-related nuclear cataracts in monozygotic and dizygotic twins. N Engl J Med.

[3] Singh R, Ram J, Kaur G, Prasad R (2012). Galactokinase deficiency induced cataracts in Indian infants: identification of 4 novel mutations in GALK gene. Curr Eye Res.

[4] Yang J, Luo J, Zhou P, Fan Q, Luo Y, Lu Y (2013). Association of the ephreceptor tyrosinekinase-type A2 (EPHA2) gene polymorphism rs 3754334 with age-related cataract risk: a meta-analysis. PLoS One.

[5] Thampi P, Hassan A, Smith JB, Abraham EC (2002). Enhanced C-terminal truncation of alphaA- and alphaB-crystallins in diabetic lenses. Invest Ophthalmol Vis Sci.

[6] Andley UP (2007). Crystallins in the eye: function and pathology. Prog Retin Eye Res.

[7] Chen Y, Yi L, Yan GQ, Jang YX, Fang YW, Wu XH, Zhou XW, Wei LM (2010). Decreased chaperone activity of alpha-crystallins in naphthalene-induced cataract possibly results from C-terminal truncation. J Int Med Res.

[8] Zhou P, Luo Y, Liu X, Fan L, Lu Y (2012). Down-regulation and CpG island hypermethylation of CRYAA in age-related nuclear cataract. FASEB J.

[9] Horwitz J (2003). Alpha-crystallin. Exp Eye Res.

[10] Andley UP (2009). Effects of alpha-crystallin on lens cell function and cataract pathology. Curr Mol Med.

[11] Reynolds PR (2002). In sickness and in health: the importance of translational regulation. Arch Dis Child.

[12] Chavez-Mardones J, Valenzuela-Munoz V, Nunez-Acuna G, Maldonado-Aguayo W, Gallardo-Escarate C (2013). Concholepas concholepas Ferritin H-like subunit (CcFer): molecular characterization and single nucleotide polymorphism associated to innate immune response. Fish Shellfish Immunol.

[13] Zuercher J, Neidhardt J, Magyar I, Labs S, Moore AT, Tanner FC, Waseem N, Schorderet DF, Munier FL, Bhattacharya S (2010). Alterations of the 5’untranslated region of SLC16A12 lead to age-related cataract. Invest Ophthalmol Vis Sci.

[14] Ma X, Jiao X, Ma Z, Hejtmancik JF (2016). Polymorphism rs7278468 is associated with age-related cataract through decreasing transcriptional activity of the CRYAA promoter. Sci Rep.

[15] Shi YY, He L (2005). SHEsis, a powerful software platform for analyses of linkage disequilibrium, haplotype construction, and genetic association at polymorphism loci. Cell Res.

[16] Bailey TL, Boden M, Buske FA, Frith M, Grant CE, Clementi L, Ren J, Li WW, Noble WS (2009). MEME SUITE: tools for motif discovery and searching. Nucleic Acids Res.

[17] Guven M, Unal M, Sarici A, Ozaydin A, Batar B, Devranoglu K (2007). Glutathione-S-transferase M1 and T1 genetic polymorphisms and the risk of cataract development: a study in the Turkish population. Curr Eye Res.

[18] Juronen E, Tasa G, Veromann S, Parts L, Tiidla A, Pulges R, Panov A, Soovere L, Koka K, Mikelsaar AV (2000). Polymorphic glutathione S-transferases as genetic risk factors for senile cortical cataract in Estonians. Invest Ophthalmol Vis Sci.

[19] Alberti G, Oguni M, Podgor M, Sperduto RD, Tomarev S, Grassi C, Williams S, Kaiser-Kupfer M, Maraini G, Hejtmancik JF (1996). Glutathione S-transferase M1 genotype and age-related cataracts. Lack of association in an Italian population. Invest Ophthalmol Vis Sci.

[20] Klein BE, Klein R, Lee KE, Gangnon RE (2008). Incidence of age-related cataract over a 15-year interval the beaver dam eye study. Ophthalmology.

[21] Xu L, Cui T, Zhang S, Sun B, Zheng Y, Hu A, Li J, Ma K, Jonas JB (2006). Prevalence and risk factors of lens opacities in urban and rural Chinese in Beijing. Ophthalmology.

[22] Brady JP, Garland D, Duglas-Tabor Y, Robison WG, Groome A, Wawrousek EF (1997). Targeted disruption of the mouse alpha A-crystallin gene induces cataract and cytoplasmic inclusion bodies containing the small heat shock protein alpha B-crystallin. Proc Natl Acad Sci U S A.

[23] Seshadri C, Shenoy M, Wells RD, Hensley-McBain T, Andersen-Nissen E, McElrath MJ, Cheng TY, Moody DB, Hawn TR (2013). Human CD1a deficiency is common and genetically regulated. J Immunol.

[24] Suske G (1999). The sp-family of transcription factors. Gene.

[25] Li L, He S, Sun JM, Davie JR (2004). Gene regulation by Sp1 and Sp3. Biochem Cell Biol.

[26] Li L, Davie JR (2010). The role of Sp1 and Sp3 in normal and cancer cell biology. Ann Anat.

[27] Liu X, Zhou P, Fan F, Li D, Wu J, Lu Y, Luo Y (2016). CpG site methylation in CRYAA promoter affect transcription factor Sp1 binding in human lens epithelial cells. BMC Ophthalmol.

