# Supplementary material for: Association of alpha A-crystallin polymorphisms with susceptibility to nuclear age-related cataract in a Han Chinese population
Source: BMC Ophthalmol. 2017 Jul 29;17:133. doi: 10.1186/s12886-017-0529-9 (PMC5534246; doi:10.1186/s12886-017-0529-9)
Supplement: Supplementary file 1 — The transcription factor binding sites overlapping rs7278468. This is the raw data of the in-silico analysis. (DOCX 52 kb) [file 12886_2017_529_MOESM1_ESM.docx]

**Association of Alpha A-crystallin Polymorphisms with Susceptibility to Nuclear Age-Related Cataract in a Han Chinese population**

| #pattern name | TF name | binding site start | binding site stop | binding DNA strand | sequence name | score | p-value | q-value | matched sequence |
| --- | --- | --- | --- | --- | --- | --- | --- | --- | --- |
| MA0741.1 | KLF16 | 382 | 392 | - | cryaa_promoter | 14.5862 | 6.57E-06 | 0.0228 | CACACACCCCC |
| MA0746.1 | SP3 | 382 | 392 | - | cryaa_promoter | 13.3659 | 1.16E-05 | 0.0415 | CACACACCCCC |
| MA0139.1 | CTCF | 382 | 400 | - | cryaa_promoter | 8.57377 | 7.13E-05 | 0.0923 | TCGCCAGAGGAGGGAGAGC |
| MA0471.1 | E2F6 | 381 | 391 | - | cryaa_promoter | 13.7069 | 1.74E-05 | 0.0639 | AGGAGGGAGAG |
| MA0516.1 | SP2 | 379 | 393 | + | cryaa_promoter | 12.2931 | 2.64E-05 | 0.0498 | CTCCCTCCTCTGGCG |
| MA0079.3 | SP1 | 379 | 389 | + | cryaa_promoter | 11.5192 | 5.14E-05 | 0.0647 | CTCCCTCCTCT |

Table S1. Transcription factor binding sites overlapping rs7278468
