# Supplementary material for: Association of alpha A-crystallin polymorphisms with susceptibility to nuclear age-related cataract in a Han Chinese population
Source: BMC Ophthalmol. 2017 Jul 29;17:133. doi: 10.1186/s12886-017-0529-9 (PMC5534246; doi:10.1186/s12886-017-0529-9)
Supplement: Supplementary file 2 — The whole gels of the NC and Sp1 ChIPs respectively. This is the raw data of the chromatin immunoprecipitation (ChIP) analysis described above. (DOCX 645 kb) [file 12886_2017_529_MOESM2_ESM.docx]

**Association of Alpha A-crystallin Polymorphisms with Susceptibility to Nuclear Age-Related Cataract in a Han Chinese population**


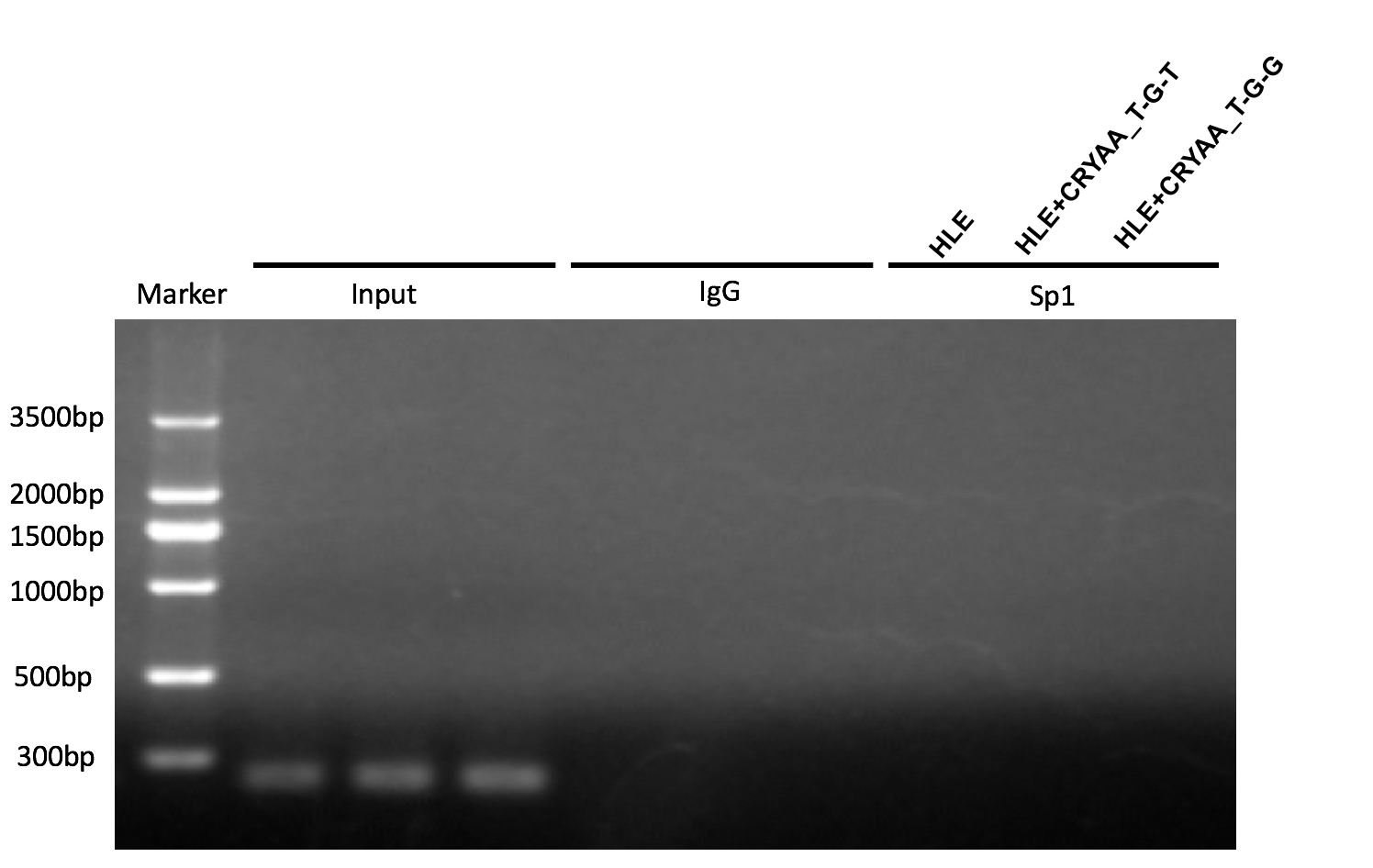


Figure S1. NC ChIP whole gel.


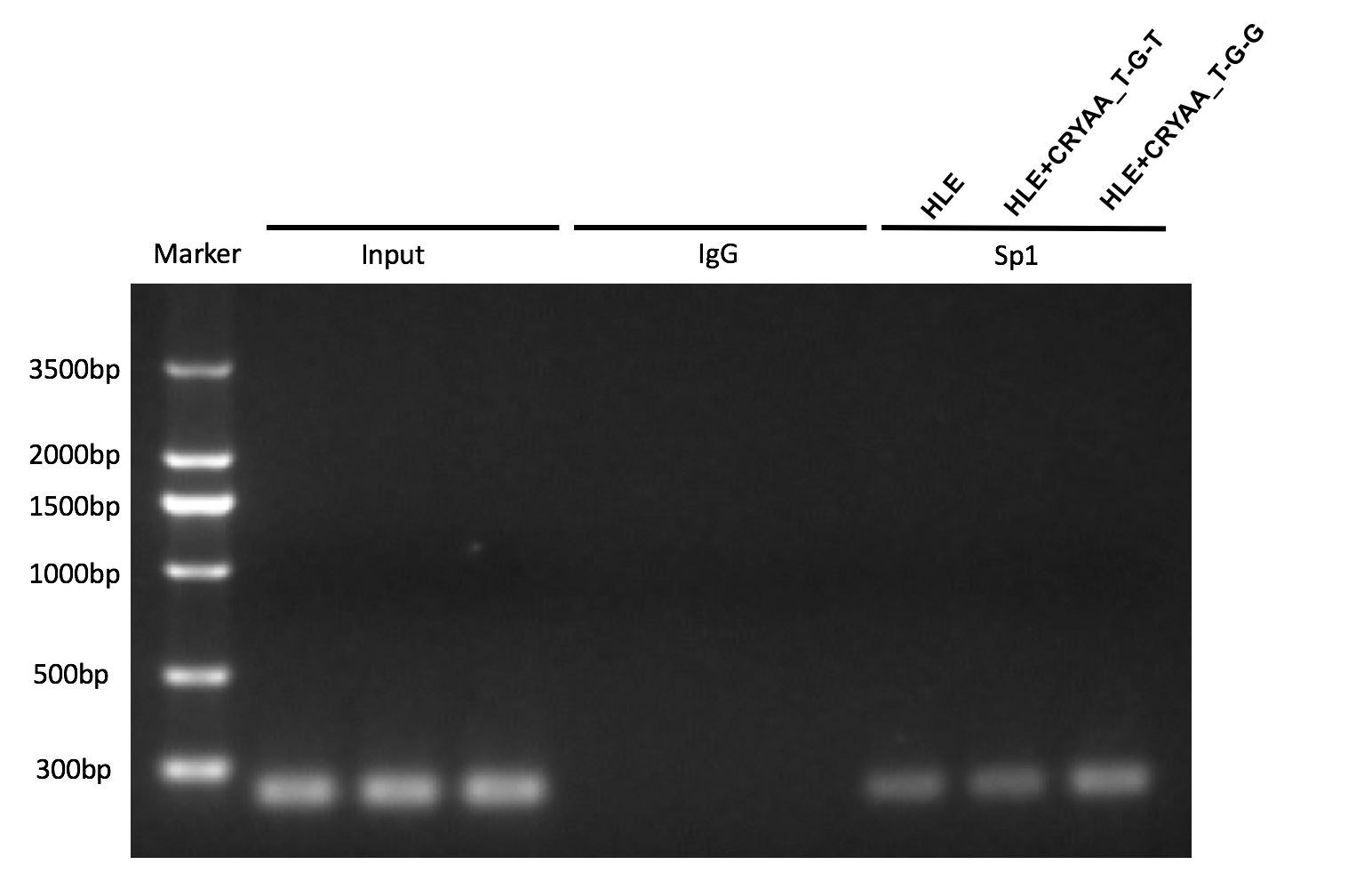


Figure S2. Sp1 ChIP whole gel.
